# An aberrantly sustained emergency granulopoiesis response accelerates postchemotherapy relapse in *MLL1*-rearranged acute myeloid leukemia in mice

**DOI:** 10.1074/jbc.RA120.013206

**Published:** 2020-05-28

**Authors:** Hao Wang, Chirag A. Shah, Liping Hu, Weiqi Huang, Leonidas C. Platanias, Elizabeth A. Eklund

**Affiliations:** 1 Department of Medicine, Northwestern University, Chicago, Illinois, USA; 2 Jesse Brown Veterans Affairs Medical Center, Chicago, Illinois, USA

**Keywords:** leukemia, gene regulation, innate immunity, ubiquitin ligase, receptor tyrosine kinase (RTK), mixed lineage leukemia (MLL), emergency granulopoiesis, cancer chemotherapy, kinase signaling, TRIAD1, leukemia, E3 ubiquitin ligase

## Abstract

Acute myeloid leukemia (AML) with mixed lineage leukemia 1 (*MLL1*) gene rearrangement is characterized by increased expression of a set of homeodomain transcription factors, including homeobox A9 (HOXA9) and HOXA10. The target genes for these regulators include fibroblast growth factor 2 (*FGF2*) and Ariadne RBR E3 ubiquitin ligase 2 (*ARIH2*). FGF2 induces leukemia stem cell expansion in MLL1-rearranged AML. ARIH2 encodes TRIAD1, an E3 ubiquitin ligase required for termination of emergency granulopoiesis and leukemia suppressor function in *MLL1*-rearranged AML. Receptor tyrosine kinases (RTKs), including the FGF receptor, are TRIAD1 substrates that are possibly relevant to these activities. Using transcriptome analysis, we found increased activity of innate immune response pathways and RTK signaling in bone marrow progenitors from mice with *MLL1*-rearranged AML. We hypothesized that sustained RTK signaling, because of decreased TRIAD1 activity, impairs termination of emergency granulopoiesis during the innate immune response and contributes to leukemogenesis in this AML subtype. Consistent with this, we found aberrantly sustained emergency granulopoiesis in a murine model of *MLL1*-rearranged AML, associated with accelerated leukemogenesis. Treating these mice with an inhibitor of TRIAD1-substrate RTKs terminated emergency granulopoiesis, delayed leukemogenesis during emergency granulopoiesis, and normalized innate immune responses when combined with chemotherapy. Emergency granulopoiesis also hastened postchemotherapy relapse in mice with *MLL1*-rearranged AML, but remission was sustained by ongoing RTK inhibition. Our findings suggest that the physiological stress of infectious challenges may drive AML progression in molecularly defined subsets and identify RTK inhibition as a potential therapeutic approach to counteract this process.

Emergency (stress) granulopoiesis is the process for episodic granulocyte production in response to infectious challenge, a fundamental aspect of the innate immune response (1–3). In contrast, steady-state granulopoiesis is a continuous process for replacing granulocytes lost to normal programed cell death (1). Initiation of emergency granulopoiesis requires IL1β, which induces a 10-fold increase in G-CSF relative to steady state (1–6). Genotoxic stress is increased during emergency granulopoiesis because of shortened S phase, accelerated differentiation, and reactive oxygen species produced by accumulating bone marrow granulocytes.

We that found termination of emergency granulopoiesis requires the E3 ubiquitin ligase TRIAD1 (5). TRIAD1 mediates endosomal degradation (*versus* recycling) of various receptors, including FGF-R, platelet-derived growth factor receptor (PDGF-R), vascular endothelial growth factor receptor (VEGF-R), and αv integrin (5, 7–9). TRIAD1 increases during granulopoiesis, and engineered overexpression of TRIAD1 in bone marrow progenitors decreases colony formation and impairs the proliferative response to cytokines, including G-CSF (7, 8). The *ARIH2* promoter is repressed by HOXA9 in hematopoietic stem cells (HSCs) and progenitor cells but activated by HOXA10 in differentiating/mature phagocytes (5, 7). This suggests HOX proteins regulate RTK signaling and emergency granulopoiesis via TRIAD1.

An adverse prognosis subtype of AML is defined by increased expression of homeodomain transcription factors, including HOXB3, B4, A7-11, MEIS1, and rapid relapse after standard chemotherapy. This includes AML with *MLL1* gene rearrangements, *MYST3-CREBBP* gene translocation, or an adverse prognosis subset with normal karyotype (10–15). *MLL1* oncoproteins aberrantly recruit epigenetic modifiers to *HOX* promoters, but mechanisms for HOX overexpression in other subtypes are unknown (16, 17).

HOXA9 and HOXA10 cooperate to activate genes that enhance HSC and progenitor expansion, including FGF2 and β3 INTEGRIN genes (7, 18–23). We found HOXA9/HOXA10-dependent, autocrine production of FGF2 by bone marrow progenitors expressing *MLL1* oncoproteins, resulting in hypersensitivity to cytokines that activate phosphoinositol 3-kinase (PI3K) (18, 19, 23). HOXA9/HOXA10 also induced αvβ3 integrin expression and enhanced proliferation via Syk in these cells (22). Because FGF-R and αv are TRIAD1 substrates, these receptors may be regulated by a balance of HOXA9 *versus* HOXA10 activities. We found TRIAD1 progressively decreased during leukemogenesis in mice with *MLL1*-rearranged AML, and TRIAD1 knockdown accelerated leukemogenesis in these mice (24).

A set of phagocyte effector genes are activated by HOXA9 during granulopoiesis but repressed by HOXA10 (5, 25–27). This suggests that phenotypic differentiation is promoted by HOXA9 but impeded by HOXA10. Consistent with this, *HOXA9*^−/−^ mice exhibit impaired G-CSF–induced granulocytosis, but emergency granulopoiesis is enhanced and sustained in *HOXA10*^−/−^ mice (5, 28). We found that re-expressing TRIAD1 in *HOXA10*^−/−^ bone marrow rescued this phenotype, suggesting a role for degradation of TRIAD1 substrates in terminating the innate immune response (5).

For the current work, we found increased activity of innate immune response pathways and RTK signaling in bone marrow progenitors from mice with *MLL1*-rearranged AML compared with control mice. We investigated the functional significance of these findings by determining the impact of emergency granulopoiesis on leukemogenesis in mice with *MLL1*-rearranged AML. Based on the possibility that TRIAD1 contributes to terminating the innate immune response via RTK degradation, we tested the impact of inhibiting TRIAD1-RTK substrates on AML progression and postchemotherapy relapse in this molecular subtype. We hypothesized that RTK inhibition would protect leukemia stem cells (LSCs) from the stress of infectious challenge and improve outcomes.

## Results

### Innate immune response pathways, RTK signaling, and RAP1 are activated in MLL1-ELL–induced AML

Mice that are transplanted with *MLL1* oncoprotein–transduced bone marrow develop transplantable AML after several months, suggesting that additional mutations are required (24, 29). To generate a population of mice with established disease for molecular characterization, recipients of MLL1-ELL–transduced, syngeneic bone marrow were sacrificed upon development of AML (circulating myeloid blasts of >30% of white blood cells or 15,000/mm^3^), and bone marrow was transplanted into secondary recipients. Eight weeks after secondary transplant, we collected LIN^−^ bone marrow cells for comparison with LIN^−^ cells from recipients of control vector-transduced bone marrow. RNA-sequencing (RNA-Seq)) and gene ontology analyses were performed. The goal of this experiment was to identify genes or pathways that contribute to leukemogenesis in HOX-overexpressing AML. The plan was to assess select candidates for a functional contribution to leukemogenesis and the potential for therapeutic targeting.

**Figure 1.**
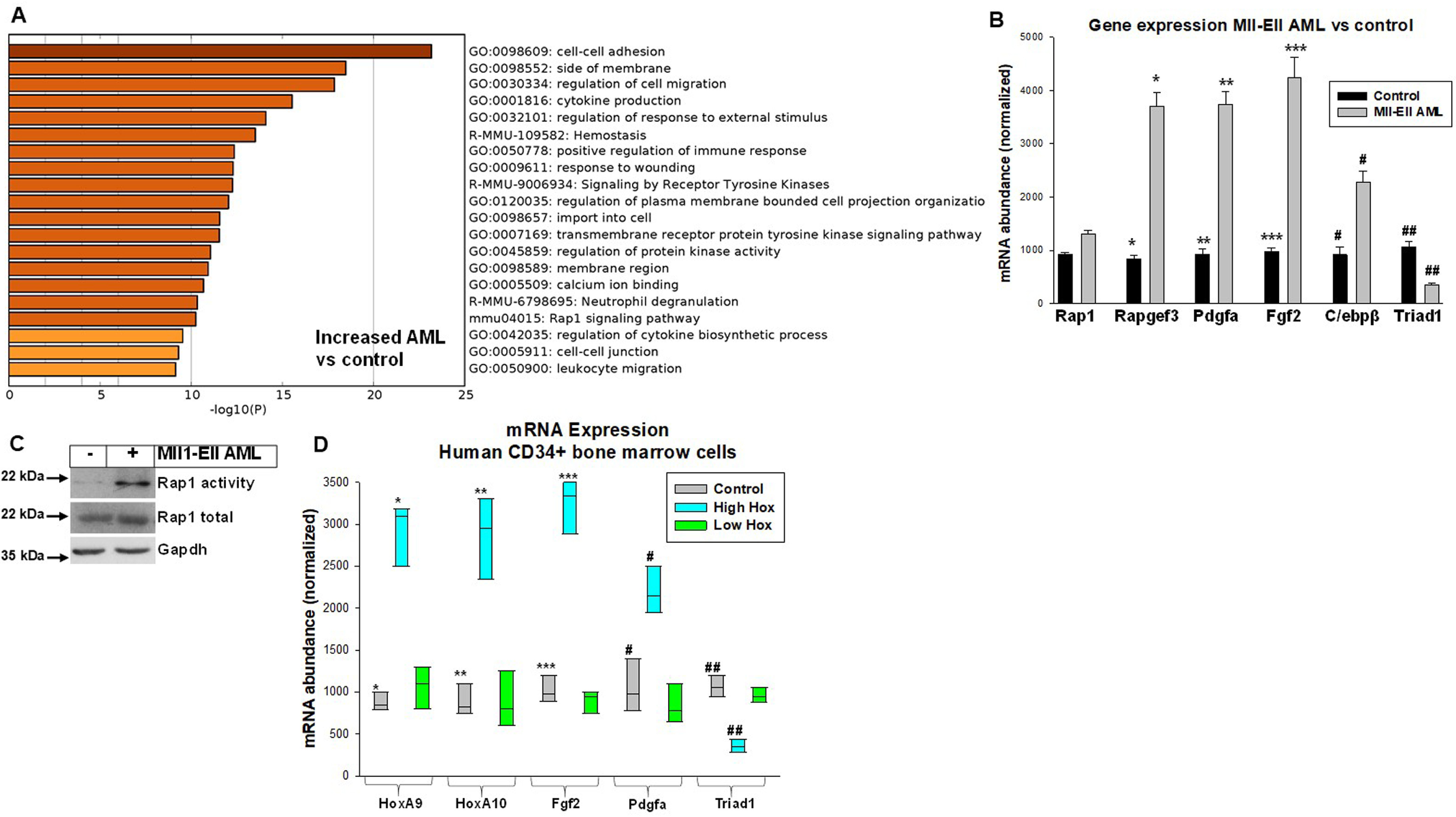
**Transcriptome analysis identified activation of pathways involved in the innate immune response, RTK signaling, and RAP1 signaling in MLL1-ELL–induced AML.** Secondary recipients of bone marrow from mice with established MLL1-ELL-AML were compared with control mice. *A*, gene ontology analysis associated AML with activation of pathways involved in positive regulation of the innate immune response, RTK signaling, and RAP1 activity. LIN^−^ bone marrow cells were analyzed by RNA-Seq. *B*, changes in expression of genes involved in these processes were confirmed in LIN^−^CKIT^+^ cells from mice with MLL1-ELL–induced AML *versus* control mice. Gene expression was studied by quantitative, real-time PCR. Statistically significant differences are indicated by *, **, ***, #, ##, and ### (*p* < 0.001, *n* = 4). *C*, RAP1 activity was increased in LIN^−^CKIT^+^ cells from mice with AML. Activity was assayed by affinity to RAL GDS RBD (GAPDH as a loading control). *D*, increased HOX expression in human LIN^−^CD34^+^ AML cells correlated with altered expression of target genes FGF2, PDGFA, and TRIAD1. Quantitative PCR was performed. Statistically significant differences are indicated by *, **, ***, #, and ## (*p* < 0.001, *n* = 4).

We found increased activity of pathways involved in positive regulation of the innate immune response, transmembrane RTK signaling, and RAP1 signaling in mice with AML compared with control mice (Fig. 1*A*). Consistent with previous expression profiles, HOX and MEIS mRNAs were increased in *MLL1*-rearranged AML (12, 13). Expression of PDGFA and various FGFs was also increased, but TRIAD1 and 2 were decreased. Increased RTK ligands, combined with impaired degradation of RTKs by TRIAD1, might sustain the innate immune response, and RAP1 is activated by RTKs, including FGF-R and PDGF-R.

We verified some of the differences in gene expression identified by RNA-Seq in independent experiments with LIN^−^CKIT^+^ bone marrow cells from mice with AML or control mice. We found increased FGF2 and decreased TRIAD1 in AML, consistent with our prior studies (*p* < 0.001, *n* = 4). We also found increased expression of PDGFA and RAP1 regulatory genes, but not RAP1 (*p* < 0.01, *n* = 4) (Fig. 1*B*). Increased RAP1 signaling was confirmed by activity assay (Fig. 1*C*). Activation of immune response and RTK signaling pathways were addressed in the remainder of the studies.

In human LIN^−^CD34^+^ AML cells, we previously correlated increased HOXA9 and HOXA10 expression with autocrine production of FGF2 and decreased TRIAD1 (18, 24). In the current study, we grouped LIN^−^CD34^+^ cells from human AML subjects by HOXA9 and HOXA10 expression relative to control LIN^−^CD34^+^ cells. We found samples with high HOX expression (>2 S.D. above control mean) had increased PDGFA and FGF2 but decreased TRIAD1 compared with control samples or samples from subjects without increased HOX expression (≤2 S.D. above control) (*n* = 4, *p* < 0.001) (Fig. 1*D*). This was consistent with results of murine studies.

### Emergency granulopoiesis accelerated leukemogenesis in MLL1-rearranged AML in an RTK-dependent manner

Activation of innate immune response pathways in MLL1-ELL-AML bone marrow progenitors could be due to mutations that constitutively activate inflammatory pathways or those that impair inactivation of a physiologic immune response. To investigate the latter, we induced emergency granulopoiesis in primary recipients of MLL1-ELL–transduced bone marrow (or control mice) by intraperitoneal injection of alum (ovalbumin/aluminum chloride IP) (2–4). This antigen/adjuvant combination induces an IL1β-dependent response that is similar to live pathogens but without death or chronic infection in the mice (2, 3). Other cohorts were injected with saline as a steady-state control. Injections began 8 weeks after transplant, prior to development of overt AML, and were repeated every 4 weeks to mimic repeated infectious challenge.

**Figure 2.**
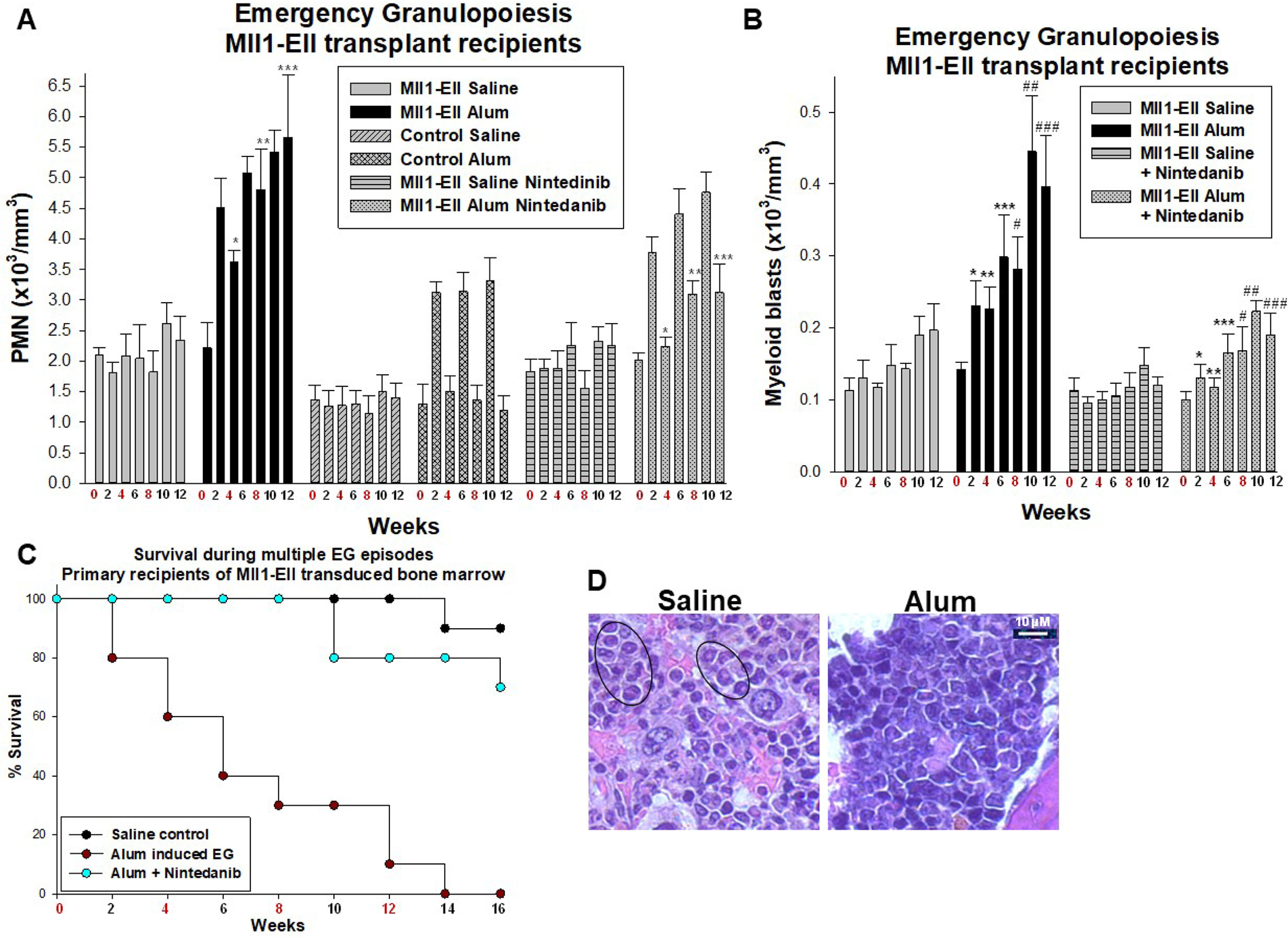
**Emergency granulopoiesis accelerated leukemogenesis in recipients of MLL1-ELL–transduced bone marrow.** The mice were transplanted with MLL1-ELL–transduced or control bone marrow and injected with alum every 4 weeks to stimulate emergency granulopoiesis (*EG*) or saline as a steady-state control (injection weeks indicated in *red*). Some cohorts were treated daily with an RTK inhibitor (nintedanib). *A*, circulating granulocytes (PMNs) returned to steady-state levels after alum injection in control mice, but not in recipients of MLL1-ELL–transduced bone marrow. The latter was improved by RTK inhibition. Statistically significant differences are indicated by *, **, and *** (*p* < 0.001, *n* = 6). *B*, alum-injected recipients of MLL1-ELL–transduced bone marrow had rapidly increasing circulating myeloid blasts *versus* those treated with RTK inhibitor or at steady state. Statistically significant differences are indicated by *, **, ***, #, ##, and ### (*p* < 0.002, *n* = 6). *C*, alum injection shortened survival in recipients of MLL1-ELL–transduced bone marrow compared with mice at steady state, but this was improved by RTK inhibition (p < 0.001, *n* = 6). *D*, bone marrow blasts appeared earlier in alum-injected recipients of MLL1-ELL–transduced bone marrow compared with steady state. Sternal bone marrow examined 2 weeks after the second alum or saline injection (hematoxylin and eosin stain, 40× magnification) revealed extensive involvement with myeloid blasts in alum-treated mice but only scattered blasts in steady-state control (*circled*). *PMN*, polymorphonuclear leukocyte.

In WT mice, circulating granulocytes were maximal 2 weeks after alum injection and returned to steady-state levels by 4 weeks (Fig. 2*A*) (4, 5). The percentage of increase in circulating granulocytes after the first alum injection was equivalent in control mice and recipients of MLL1-ELL–transduced bone marrow (∼2-fold), although the baseline was higher in the latter. However, circulating granulocytes did not return to baseline after alum injection in mice with MLL1-ELL–transduced bone marrow. Each subsequent alum injection further increased circulating granulocytes in these mice, resulting in relative granulocytosis 4 weeks after the first injection compared with mice at steady state (*p* < 0.001, *n* = 6) (Fig. 2*A*).

Alum-injected recipients of MLL1-ELL–transduced bone marrow had increased circulating myeloid blasts by 2 weeks (*p* < 0.001, *n* = 6) (Fig. 2*B*) and shorter survival (*p* < 0.001, *n* = 9) (Fig. 2*C*) compared with mice at steady state. The bone marrow was infiltrated by myeloid blasts in alum-injected recipients of MLL1-ELL–transduced bone marrow at a time point when only scattered clusters of blasts appeared at steady state (Fig. 2*D*). Blasts were not seen in WT mice at any point during the experiment (4, 5).

Because we found activation of RTK signaling and decreased TRIAD1 expression in mice with *MLL1*-rearranged AML, we hypothesized that degradation of TRIAD1-substrate RTKs contributes to terminating emergency granulopoiesis. To test this, we treated primary recipients of MLL1-ELL–transduced bone marrow with nintedanib, an inhibitor of TRIAD1-substrate RTKs, including FGF-R, PDGF-R, and VEGF-R (30). FGF-R and PDGF-R are expressed on HSC or AML cells (18, 24, 31).

Cohorts of mice were treated daily with nintedanib, starting at the first alum injection. We found improved resolution of emergency granulopoiesis in recipients of MLL1-ELL–transduced bone marrow that were treated with RTK inhibitor *versus* untreated cohorts (*p* < 0.001, *n* = 9), although granulocytes still rose relative to steady state (Fig. 2*A*). RTK inhibition delayed emergence of circulating myeloid blasts during emergency granulopoiesis (*p* < 0.01, *n* = 9) (Fig. 2*B*) and improved survival (*p* < 0.001, *n* = 9) (Fig. 2*C*).

**Figure 3.**
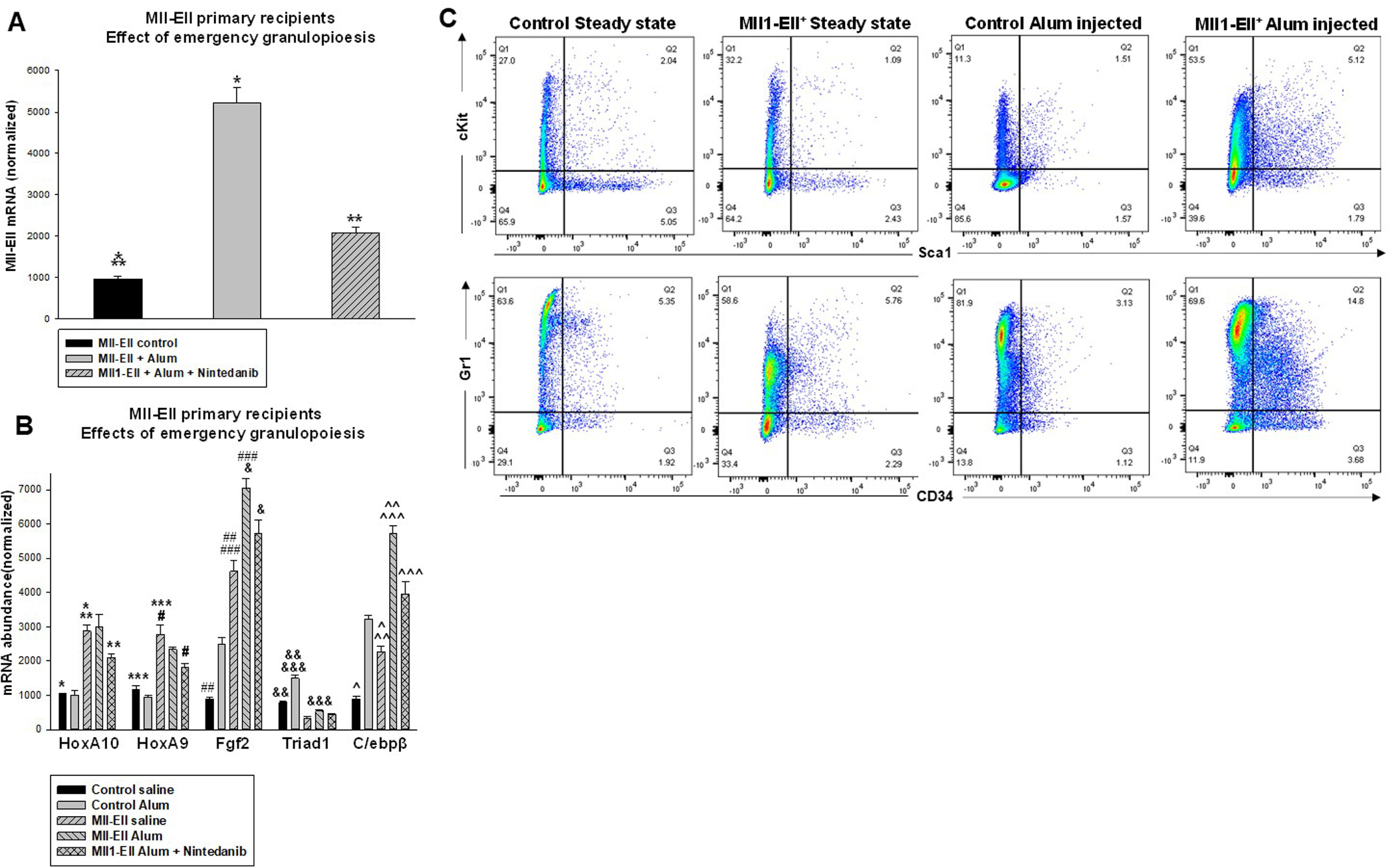
**Emergency granulopoiesis induces leukemia cell expansion in recipients of MLL1-ELL–transduced bone marrow and modulates expression of genes that regulate this process.** The mice were transplanted with MLL1-ELL–transduced or control bone marrow and injected either with alum every 4 weeks to stimulate emergency granulopoiesis or with saline as a steady-state control. Some cohorts were treated daily with an RTK inhibitor (nintedanib). The mice were sacrificed for analysis 2 weeks after the second injection. *A*, MLL1-ELL transcripts in the bone marrow increased during emergency granulopoiesis, but RTK inhibition decreased this effect. Bone marrow LIN^−^CKIT^+^ cells were analyzed by quantitative PCR for MLL1-ELL fusion transcripts. Statistically significant differences are indicated by *, **, and *** (*p* < 0.01, *n* = 4). *B*, expression of FGF2 and C/EBPβ was increased in MLL1-ELL bone marrow recipients compared with control mice. This was enhanced by emergency granulopoiesis but impaired by RTK inhibitor. LIN^−^CKIT^+^ bone marrow cells were analyzed by quantitative PCR for HOX genes, FGF2, TRIAD1, or C/EBPβ. Statistically significant differences are indicated by *, **, ***, #, ##, ###, &, &&, &&&, ^, ^^, and ^^^ (*p* < 0.02, *n* = 4). *C*, SCA1^+^CKIT^+^ bone marrow cells expand during emergency granulopiesis in recipients of MLL1-ELL–transduced bone marrow but contract in control mice. This was associated with differentiation block, as indicated by relative expansion of CD34^+^GR1^+^ cells *versus* CD34^−^GR1^+^ cells in control mice. Representative histograms are shown.

To clarify the effects of emergency granulopoiesis on leukemogenesis, the mice were studied 2 weeks after the second alum or saline injection. We found expansion of AML cells during emergency granulopoiesis compared with steady state, as indicated by MLL1-ELL fusion transcript abundance in LIN^−^CKIT^+^ bone marrow cells (*p* < 0.001, *n* = 4) (Fig. 3*A*). This effect was decreased by RTK-inhibitor treatment (*p* < 0.001, *n* = 4).

To further characterize this process, we studied emergency granulopoiesis-associated genes in these cells. C/EBPβ is required to initiate, and TRIAD1 to terminate, emergency granulopoiesis. We found that C/EBPβ was increased at baseline in recipients of MLL1-ELL–transduced bone marrow *versus* control recipients and also 2 weeks after alum injection (*p* < 0.01, *n* = 4) (Fig. 3*B*). TRIAD1 induction during emergency granulopoiesis was less in mice with MLL1-ELL–transduced bone marrow compared with control mice (*p* < 0.01, *n* = 4). Alum increased FGF2 in both groups, but expression was greater in recipients of MLL1-ELL–transduced bone marrow (*p* < 0.001, *n* = 4). RTK-inhibitor treatment impaired the alum-induced increase in C/EBPβ and FGF2 in mice with MLL1-ELL–transduced bone marrow (*p* < 0.01, *n* = 4), but TRIAD1 was not altered.

HOXA9 and HOXA10 were increased LIN^−^CKIT^+^ cells from mice with MLL1-ELL–transduced bone marrow compared with control mice, as anticipated (*p* < 0.001, *n* = 4). HOX expression was not altered by alum-induced emergency granulopoiesis but was decreased by RTK-inhibitor treatment. This was consistent with a positive feedforward mechanism between FGF-R activation and *HOX* transcription, previously described (32, 33).

We also compared bone marrow population distributions during emergency granulopoiesis in mice with MLL1-ELL–transduced bone marrow and control mice. 2 weeks after alum injection, we found expansion of CD34^−^GR1^+^ maturing granulocytes in control mice but contraction of immature SCA1^+^CKIT^+^ cells (4). In contrast, the latter population expanded in alum-injected mice with MLL1-ELL^+^ bone marrow, but differentiation was blocked (relative expansion of CD34^+^GR1^+^ cells *versus* CD34^−^GR1^+^ cells compared with control mice) (Fig. 3*C*).

### RTK inhibition postchemotherapy delayed relapse in mice with MLL1-rearranged AML

Mice with *MLL1*-rearranged AML achieve remission but relapse rapidly after treatment with a chemotherapy regimen similar to standard human AML therapy (34). We considered the possibility that RTK inhibition might decrease the activity of innate immune response pathways and thereby influence relapse in these mice. This would functionally associate two pathways identified by transcriptome analysis with each other and with leukemogenesis.

**Figure 4.**
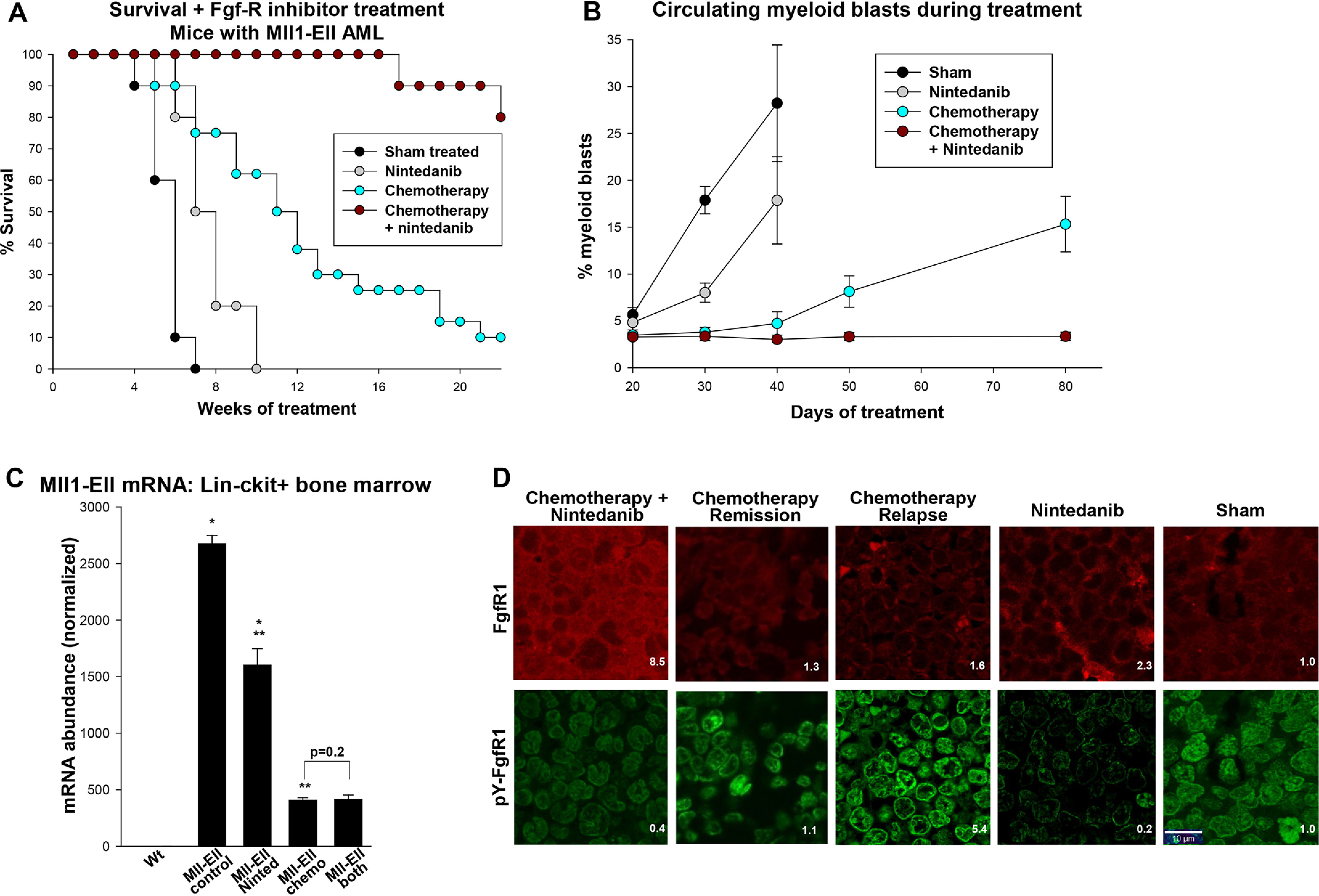
**RTK inhibition prevented postchemotherapy relapse in mice with MLL1-ELL–induced AML.** Secondary recipients of bone marrow from mice with established MLL1-ELL–induced AML were treated with cytosine arabinoside + doxorubicin (5 + 3 chemotherapy), an RTK inhibitor (nintedanib) alone, 5 + 3 chemotherapy + RTK inhibitor, or buffer control. *A*, postchemotherapy survival was significantly prolonged by RTK-inhibitor maintenance. Survival was 50% at 12 weeks postchemotherapy but was not reached at 30 weeks with the addition of nintedanib (*p* < 0.001, *n* = 10). *B*, maintenance therapy with RTK inhibitor prevented emergence of circulating myeloid blasts after chemotherapy. Statistically significant differences in circulating myeloid blasts are indicated by *, **, ***, #, and ## (*p* < 0.001, *n* = 10). *C*, MLL1-ELL transcript abundance was similar in LIN^−^CKIT^+^ cells from mice treated with chemotherapy *versus* chemotherapy + RTK inhibitor. The mice were sacrificed 8 weeks after completion of chemotherapy or 8 weeks after transplant for untreated mice. Statistically significant differences are indicated by * and ** (*p* < 0.0001, *n* = 4). *D*, phospho-FGF-R1 was increased in mice with MLL1-ELL^+^ AML but decreased by RTK-inhibitor treatment. Murine sternal bone marrow was analyzed with antibody to FGF-R1 or pY653/654-FGF-R1 (by immune-fluorescent microscopy, 40× magnification) 8 weeks after chemotherapy or 8 weeks after transplant for untreated mice. Fluorescent intensity relative to control bone marrow is indicated.

To investigate this, we studied secondary recipients of bone marrow from mice with established MLL1-ELL–induced AML. Some mice were treated with 5 days of cytosine arabinoside plus 3 days of doxorubicin (“5 + 3”) 4 weeks after transplant (34). Although survival was prolonged compared with untreated mice (*p* < 0.001, *n* = 10), all mice relapsed (Fig. 4*A*). Chemotherapy also delayed the appearance of circulating myeloid blasts compared with sham treatment (*p* < 0.001, *n* = 10) (Fig. 4*B*).

We treated other cohorts with chemotherapy plus daily RTK inhibition, with the latter continuing until death. The addition of RTK inhibitor (nintedanib) prolonged survival compared with chemotherapy alone (*p* < 0.0001, *n* = 10) (Fig. 4*A*), and circulating myeloid blasts did not appear in 20+ weeks of treatment (Fig. 4*B*). In contrast, survival in a cohort treated with RTK inhibitor alone was comparable with sham treatment, although emergence of circulating myeloid blasts was delayed (*p* < 0.01, *n* = 10).

To determine whether RTK inhibition eliminated residual LSCs after chemotherapy, we isolated LIN^−^CKIT^+^ bone marrow cells 8 weeks after therapy initiation and quantified MLL1-ELL fusion transcripts (18). Expression was a log less after chemotherapy compared with sham-treated mice (Fig. 4*C*). However, the addition of RTK inhibitor to chemotherapy did not significantly alter MLL1-ELL transcript abundance (*p* = 0.2, *n* = 4), suggesting suppression, but not elimination, of LSCs. Nintedanib treatment decreased FGF-R1 activation (phosphorylation) in the bone marrow of mice with MLL1-ELL–induced AML, although total FGF-R1 protein increased (Fig. 4*D*).

**Figure 5.**
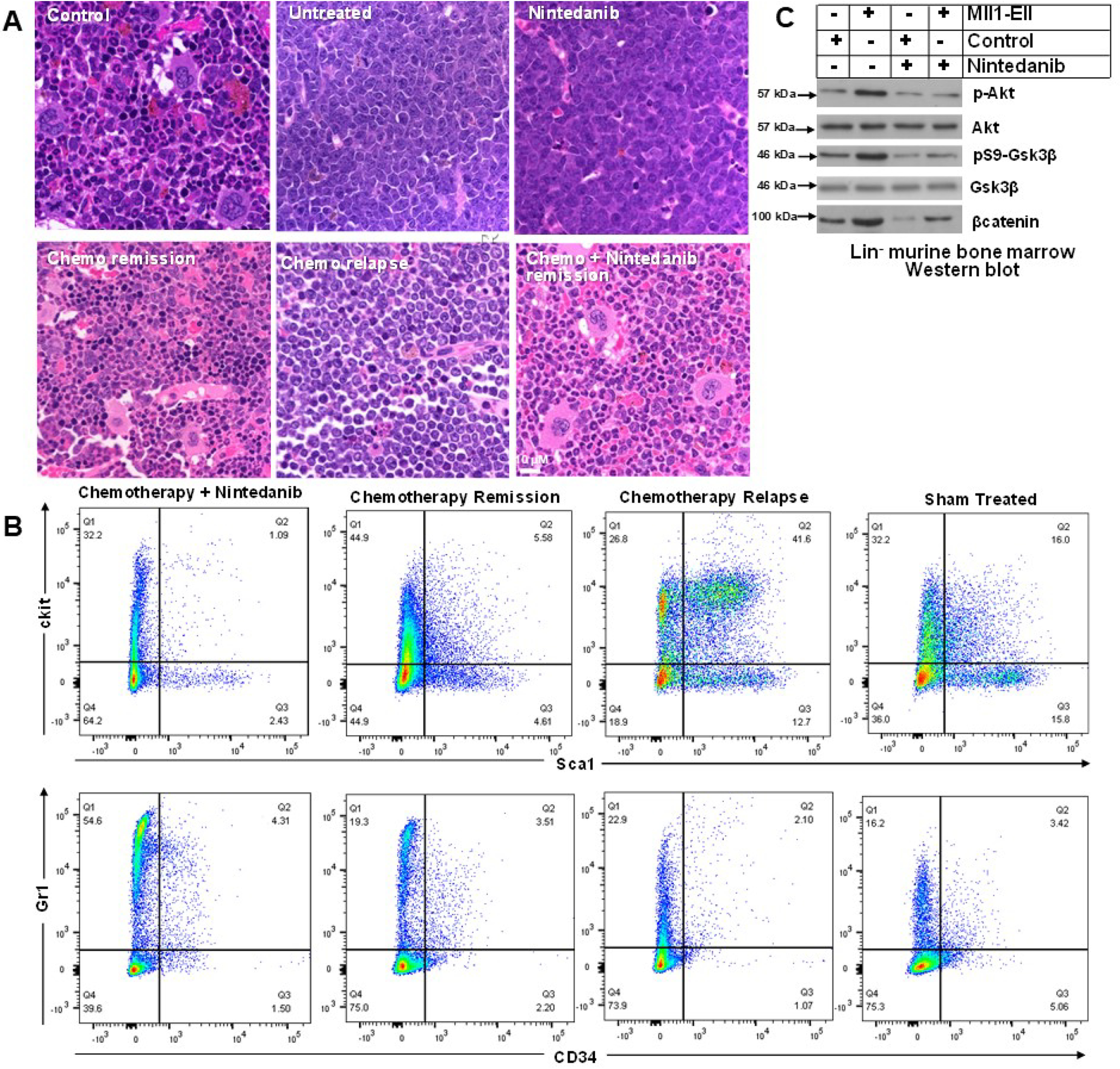
**Maintenance therapy with an RTK inhibitor (nintedanib) sustained hematologic remission after cytosine arabinoside + doxorubicin (5 + 3) chemotherapy in mice with MLL-ELL–induced AML.** Secondary recipients of bone marrow from mice with established AML were treated with 5 + 3 chemotherapy, RTK inhibitor, both, or buffer control. *A*, after 5 + 3 chemotherapy, bone marrow demonstrated hematologic remission followed by relapse, but remission was sustained by treatment with 5 + 3 chemotherapy + RTK inhibitor. Sternal bone marrow was stained with hematoxylin and eosin and examined by light microscopy (40× magnification). *B*, SCA1^+^CKIT^+^ cells were increased, and CD34^−^GR1^+^ cells were decreased, in the bone marrow of mice with MLL1-ELL-AML. These populations normalized during remission with 5 + 3 chemotherapy + RTK inhibitor but not with chemotherapy alone. *C*, PI3K pathway activity in MLL1-ELL-AML was decreased by RTK-inhibitor treatment. LIN^−^ bone marrow cells were analyzed by Western blotting for total or phospho (active) AKT, total or phospho (inactive) Gsk3β, or total β-catenin. A representative blot is shown.

Mice in hematologic remission after chemotherapy alone (Fig. 5*A*) had persistent expansion of SCA1^+^CKIT^+^ and SCA1^-^CKIT^+^ cells in the bone marrow (Fig. 5*B*), with relative decreases in CD34^−^GR1^+^ cells, compared with control mice (Fig. 3*C*). Adding RTK inhibitor to chemotherapy normalized these populations (Fig. 5*B*).

We also examined the impact of nintedanib on the PI3K pathway, activated by these RTKs. We found that this RTK inhibitor reversed activation (phosphorylation) of AKT in MLL1-ELL^+^ LIN^−^ cells, associated with inhibitory phosphorylation of GSK3β (p-S9) and destabilization of β-catenin (Fig. 5*C*).

### RTK inhibition postchemotherapy normalized innate immune response pathways in mice with MLL1-rearranged AML

**Figure 6.**
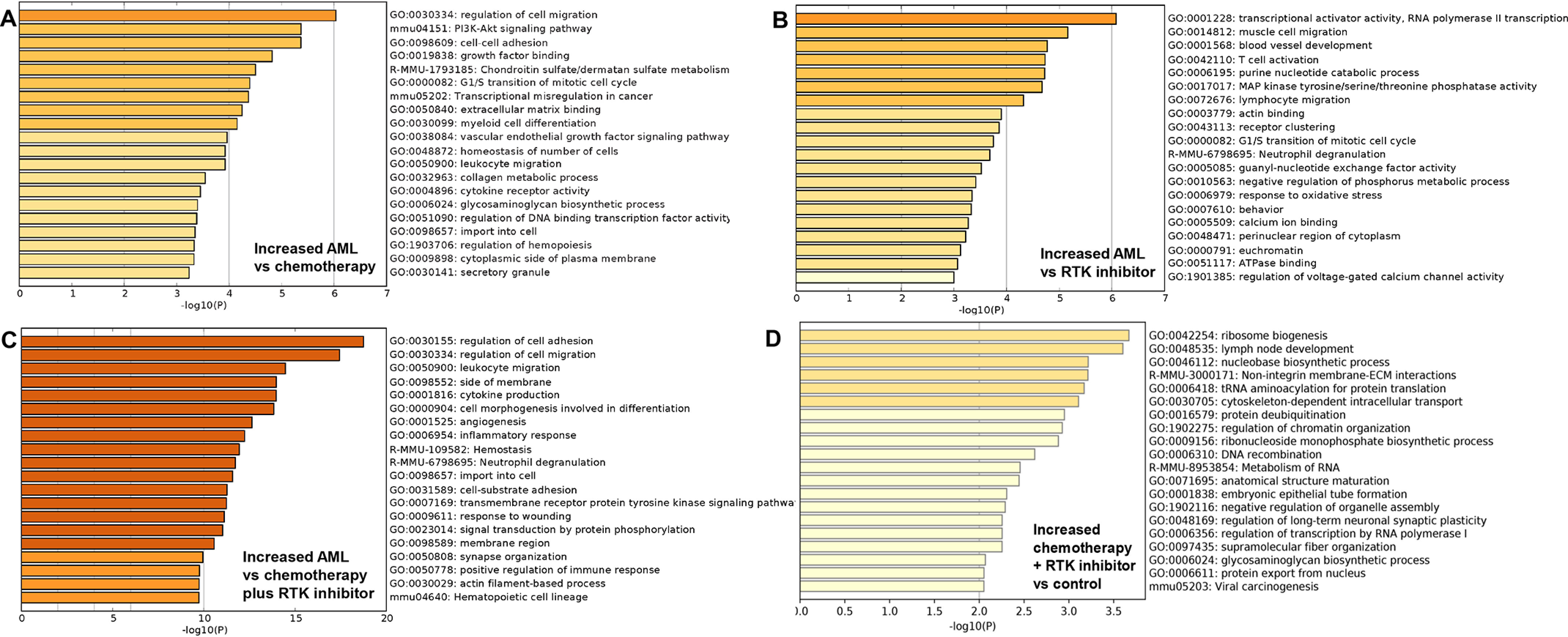
**Maintenance with an RTK inhibitor (nintedanib) after cytosine arabinoside + doxorubicin chemotherapy normalized inflammatory response and RTK signaling pathways in mice with MLL1-ELL–induced AML, but abnormalities persisted in pathways involved in ubiquitination and mRNA translation.** Secondary recipients of bone marrow from mice with established MLL1-ELL–induced AML were treated with cytosine arabinoside + doxorubicin chemotherapy (5 + 3), RTK inhibitor, both, or buffer control. RNA-Seq was performed on LIN^−^ bone marrow cells, and transcriptomes were compared (*n* = 4). The mice were sacrificed 8 weeks after chemotherapy treatment or 8 weeks after transplant for untreated mice. *A*, chemotherapy alone decreased activity of pathways involved in cytokine signaling. Differences were significantly less than comparisons between control and AML cells. *B*, treatment with nintedanib altered expression of pathways involved in guanyl-nucleotide exchange factor activity. *C* and *D*, although chemotherapy + RTK-inhibitor maintenance normalized immune response, cytokine, and RTK signaling pathways (*C*), abnormalities in mRNA translation and protein ubiquitination remained (*D*).

To investigate the contribution of RTK inhibition to activity of innate immune response pathways in *MLL1*-rearranged AML, we performed RNA-Seq of LIN^−^ bone marrow cells from secondary recipients 8 weeks after initiating treatment with chemotherapy, RTK inhibitor, or both. In mice in hematologic remission, we found that chemotherapy alone partially corrected pathways involved in cytokine receptor activity and PI3K-AKT signaling compared with sham treatment (Fig. 6*A*). Treatment with RTK inhibitor alone decreased activity in guanyl nucleotide exchange factor pathways (*i.e.* RAP1) compared with untreated AML (Fig. 6*B*). However, immune response and RTK signaling pathways normalized during postchemotherapy maintenance with nintedanib (Fig. 6*C*).

Bone marrow population distributions were similar in control mice and mice with MLL1-ELL–induced AML treated with chemotherapy plus RTK inhibitor. Transcriptomes would be identical if persistent AML cells were not contributing to the gene expression profile. In contrast, we found persistence of abnormalities in pathways involved in protein ubiquitination (consistent with decreased TRIAD1) and mRNA translation in this comparison, suggesting residual abnormalities in LIN^−^ AML populations (Fig. 6*D*).

### RTK inhibition postchemotherapy blocked emergency granulopoiesis–induced relapse in mice with MLL1-rearranged AML

Based on these results, we investigated the impact of emergency granulopoiesis on relapse after chemotherapy. Secondary recipients of bone marrow from mice with established MLL1-ELL–induced AML were treated with 5 + 3 chemotherapy with or without RTK inhibitor, as above. Emergency granulopoiesis was induced 8 weeks after therapy initiation in mice in hematologic remission (2, 35).

**Figure 7.**
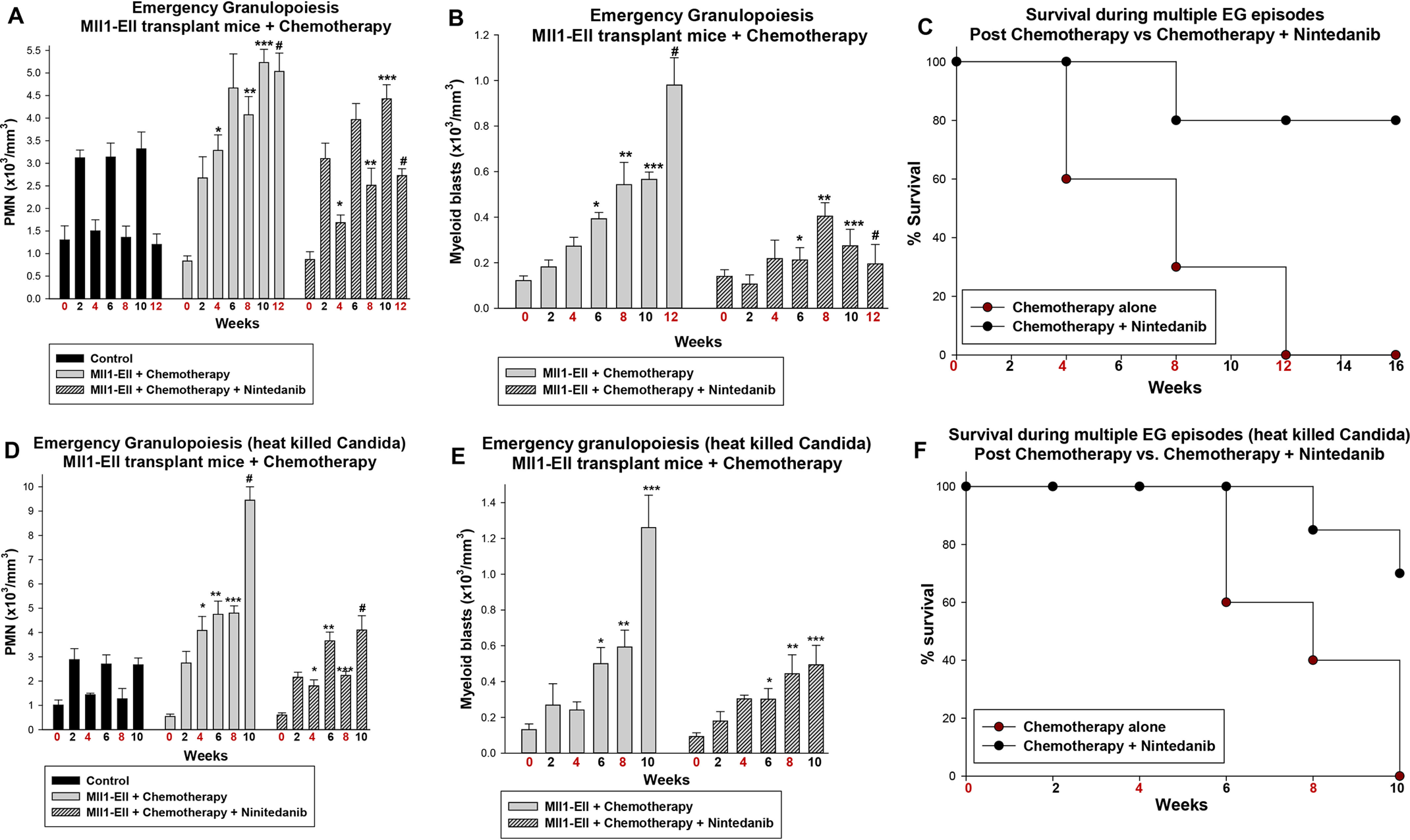
**RTK-inhibitor maintenance delayed emergency granulopoiesis–induced relapse in mice with MLL1-ELL–induced AML in chemotherapy-induced remission.** Secondary recipients of bone marrow from mice with established MLL1-ELL–induced AML were treated with cytosine arabinoside + doxorubicin (5 + 3) chemotherapy or 5 + 3 + an RTK inhibitor (nintedanib). The mice in remission were injected with alum (*A–C*) or heat-killed *Candida* (*D* and *E*) to induce emergency granulopoiesis (*n* = 6). Weeks with injections are indicated in *red*. *A* and *D*, RTK inhibition facilitated return of circulating granulocytes to steady-state levels after injection of alum or heat-killed *Candida*. Statistically significant differences are indicated by *, **, ***, and # (*p* < 0.001, *n* = 6). *B* and *E*, circulating myeloid blasts appeared later in mice treated with chemotherapy + RTK inhibitor compared with chemotherapy alone. Statistically significant differences are indicated by *, **, ***, and # (*p* < 0.01, *n* = 6). *C* and *F*, survival during episodes of alum or heat-killed *Candida*–induced emergency granulopoiesis was improved by RTK-inhibitor maintenance. Postchemotherapy survival was ∼8 weeks without RTK inhibitor but was not reached during experiments with chemotherapy + RTK inhibitor (*p* < 0.01, *n* = 6).

We found that alum injection of mice treated with chemotherapy alone resulted in granulocytosis that failed to resolve, despite normal abundance of circulating granulocytes prior to injection (Fig. 7*A*). This was associated with rapid increase in myeloid blasts (*p* < 0.001, *n* = 6) (Fig. 7*B*) and shortened survival (Fig. 7*C*) compared with mice at steady state.

Adding RTK-inhibitor maintenance improved resolution of granulocytosis after alum injection of mice in postchemotherapy remission (Fig. 7*A*). Myeloid blasts rose more slowly during repeated episodes of emergency granulopoiesis in mice treated with chemotherapy plus RTK inhibitor *versus* chemotherapy alone (*p* < 0.001, *n* = 6) (Fig. 7*B*), and survival was improved (Fig. 7*C*).

As an alternative method to stimulate emergency granulopoiesis, we injected cohorts of mice in chemotherapy-induced remission with heat-killed *Candida albicans* (35). We found a comparable granulocytosis response in WT mice injected every 4 weeks with the two stimuli (Fig. 7, *A* and *D*). Alum is relatively specific for emergency granulopoiesis, but heat-killed *Candida* is a physiologically relevant alternative.

In mice with MLL1-ELL-AML in chemotherapy-induced remission, we found that the effects of alum and heat-killed *Candida* were comparable for granulocytosis, emergence of circulating myeloid blasts (Fig. 7*E*), and survival (Fig. 7*F*). RTK-inhibitor maintenance also protected these mice from relapse and enhanced survival during multiple episodes of heat-killed *Candida* injection.

**Figure 8.**
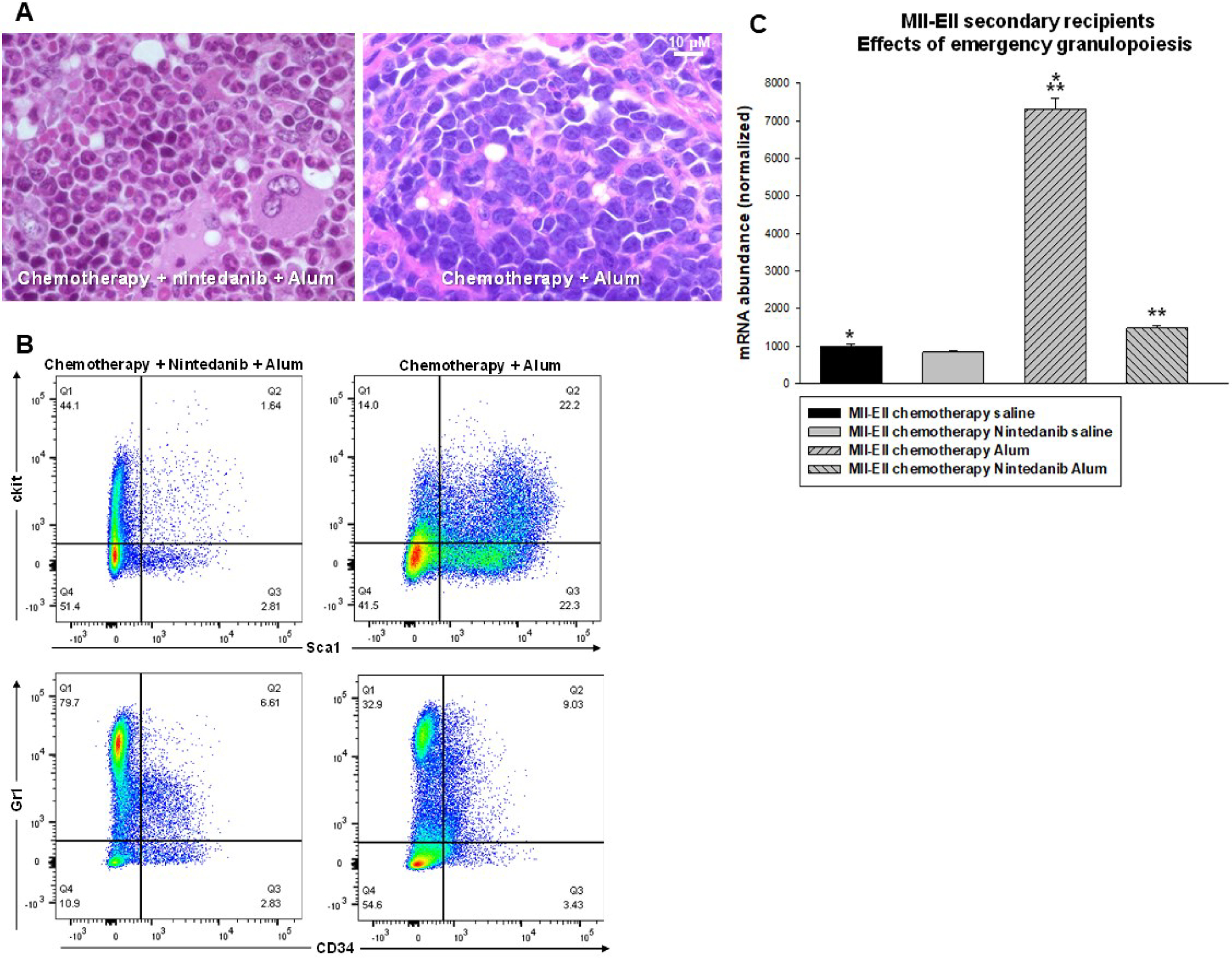
**Postchemotherapy maintenance with an RTK inhibitor (nintedanib) prevented expansion of MLL1-ELL-AML cells during episodes of emergency granulopoiesis**. Secondary recipients of bone marrow from mice with established MLL-ELL–induced AML were treated with cytosine arabinoside + doxorubicin chemotherapy (5 + 3) or 5 + 3 + an RTK inhibitor. The mice in hematologic remission were injected with alum to induce emergency granulopoiesis and analyzed 2 weeks after the second alum injection. *A*, compared with treatment with chemotherapy alone, RTK-inhibitor maintenance decreased bone marrow myeloid blasts. Sternal bone marrow was examined by hematoxylin and eosin stain (40× magnification). *B*, compared with treatment with chemotherapy alone, RTK-inhibitor maintenance prevented expansion of SCA1^+^CKIT^+^ bone marrow cells during emergency granulopoiesis and enhanced differentiating granulocytes (CD34^−^GR1^+^). *C*, compared with treatment with chemotherapy alone, RTK-inhibitor maintenance decreased MLL1-ELL transcript abundance in the bone marrow increased during episodes of emergency granulopoiesis. LIN^−^CKIT^+^ cells were analyzed by quantitative PCR. Statistical significance is indicated by * and ** (*p* ≤ 0.001, *n* = 4).

Subsequent to the second emergency granulopoiesis episode, myeloid blasts dominated the bone marrow of mice treated with chemotherapy alone (Fig. 8*A*). In contrast, the bone marrow of mice treated with chemotherapy plus RTK inhibitor exhibited granulocytosis without excess myeloid blasts at this point. Bone marrow population distribution 2 weeks after the second alum injection in mice treated with chemotherapy alone demonstrated expanded SCA1^+^CKIT^+^ cells but fewer CD34^−^GR1^+^ cells compared with chemotherapy plus RTK inhibitor (Fig. 8*B*).

We also assessed expansion of AML cells during emergency granulopoiesis by determining the abundance of MLL1-ELL fusion transcripts in LIN^−^CKIT^+^ bone marrow cells from these mice. We found that transcripts were significantly greater in alum-injected mice treated with chemotherapy alone compared with chemotherapy plus RTK inhibitor (*p* < 0.0001, *n* = 4) (Fig. 8*C*).

## Discussion

We found that episodes of emergency granulopoiesis accelerated leukemogenesis in mice transplanted with MLL1-ELL–transduced bone marrow and relapse in mice with AML in chemotherapy-induced hematologic remission. This was associated with impaired termination of this process (5, 7, 8, 24). These results supported the functional relevance of our transcriptome analysis, which identified activation of pathways involved in the innate immune response in mice with MLL1-ELL-AML compared with control mice.

We found that AML progression and postchemotherapy relapse during emergency granulopoiesis were delayed by treatment with an RTK inhibitor (nintedanib) in this murine model. We chose nintedanib because it inhibits FGF-R, PDGF-R, and VEGF-R, known TRIAD1 substrates (30). These functional results were consistent with our transcriptome findings of activated RTK signaling but decreased TRIAD1 expression in mice with MLL1-ELL-AML *versus* control mice.

We found expansion of MLL1-ELL^+^ bone marrow cells during emergency granulopoiesis, with an increase in SCA1^+^CKIT^+^ progenitors. In contrast, SCA1^+^CKIT^+^ cells contract during emergency granulopoiesis in control murine bone marrow, and expansion of mature granulocytes is relatively greater.

We previously found that FGF2 production by HOX-overexpressing LSCs resulted in autocrine stimulation *in vitro* (18, 19). In the current studies, we found increased FGF2 expression by bone marrow progenitors during emergency granulopoiesis in both control mice and those with MLL1-ELL-AML. Under normal conditions, the effects of increased FGF2 expression may be mitigated by TRIAD1-induced FGF-R degradation during termination of this process. The combination of relatively increased FGF2 and impaired TRIAD1 in MLL1-ELL-AML is anticipated to increase and sustain emergency granulopoiesis, as we found. We speculate this relative increase in FGF2 production by LSCs also influences nonclonal HSC and progenitors in these mice, but they are able to reset to steady state because of a normal increase in TRIAD1.

A relative differentiation block in LSCs may also contribute to an aberrant emergency granulopoiesis response. IL1β induces an increase in G-CSF, relative to steady state, during emergency granulopoiesis. Hypersensitivity of MLL1-ELL-LSCs to cytokines, including G-CSF, may contribute to expansion of immature populations during this process. FGF2 contributes to cytokine hypersensitivity in *MLL1*-rearranged AML, providing an additional mechanism for RTK inhibition to influence emergency granulopoiesis (18, 19).

Our results suggest inhibiting TRIAD1-substrate RTKs (with nintedanib) substitutes, to some extent, for increased TRIAD1 expression to normalize emergency granulopoiesis in MLL1-ELL-AML. Nintedanib inhibits RTKs found on HSCs and LSCs (FGF-R and PDGF-R) but also bone marrow stromal cells (FGF-R, PDGF-R, and VEGF-R). Effects of nintedanib on one or more of these receptors and the role in inhibiting LSCs *versus* modifying the bone marrow niche are of interest. This is a topic of ongoing investigation in the laboratory.

We previously found a progressive decrease in TRIAD1 expression during leukemogenesis that correlated with increased SHP1 and SHP2 (24). HOXA9 and HOXA10 are substrates for these PTPs, and *ARIH2* promoter activation requires phosphorylated HOXA10 (5, 7).

In the current study, we found RTK inhibition decreased HOXA9 or HOXA10 expression during emergency granulopoiesis. Previously, we found FGF2/FGF-R stabilizes β-catenin through the PI3K pathway in MLL1-ELL-AML (18, 19). *CDX4* is a β-catenin target gene, and CDX4 activates the *HOXA9* and *HOXA10* promoters (32, 33). RTK inhibition may block this feedforward mechanism. Other TRIAD1 substrates, such as αv integrin, are not directly influenced by RTK inhibition. Identifying such substrates and defining additional molecular targets for *MLL1*-rearranged AML are topics of interest in the laboratory.

We used alum or heat-killed *C. albicans* to induce emergency granulopoiesis. To mimic ongoing exposure of human AML patients to environmental pathogens, we studied 4-week intervals, representing the equivalent of an infection approximately every 2–3 years in human subjects (36). Our studies imply a role for RTK inhibition by TRIAD1 in preventing a relapse in the subset of AML with aberrant HOX expression and impaired management of physiologic stress.

## Materials and methods

### Oligonucleotides

Oligonucleotides were synthesized by MWG Biotech (Piedmont, NC, USA).

### Plasmid vectors

MLL1-ELL cDNA was obtained from DE Zhang (University of California, San Diego) and subcloned into the murine stem cell virus vector plasmid (Stratagene, La Jolla, CA).

### Retroviral production and murine bone marrow transplantation

Retrovirus was generated with MLL1-ELL or control murine stem cell virus vector vector in Phoenix-Ampho packaging cell line per manufacturer's instructions (Stratagene). SCA1^+^ cells were isolated from C57/BL6 murine bone marrow using a magnetic bead–linked antibody system and incubated with retroviral supernatant and Polybrene (6 μg/ml) (24). Viable cells were obtained by negative selection for annexin V. Transductions were performed with four to six independent batches of retrovirus. The packaging cell line was validated every 6 months.

### Primary and secondary murine bone marrow transplant

Lethally irradiated syngeneic mice were injected with transduced SCA1^+^ cells (2 × 10^5^), as previously described (29). Mice with established AML (WBC > 50,000, myeloid blasts >30%) were sacrificed, and total bone marrow mononuclear cells (1 × 10^6^) were transplanted into syngeneic, sublethally irradiated secondary recipients.

Primary recipients developed overwhelming AML by ∼24 weeks and secondary recipients by ∼8 weeks. Peripheral blood counts were determined every 4 weeks after transplantation using tail vein phlebotomy and an automated Hemavet counter (Erba Diagnostics, Miami, FL, USA).

### In vivo murine emergency granulopoiesis assay

To induce emergency granulopoiesis, cohorts of primary or secondary recipients or control mice were injected IP every 4 weeks with ovalbumin/aluminum chloride (referred to as alum). Alum was prepared as described, and a 0.5 ml volume was injected (2–5). For other experiments, *C. albicans* (SC5314 strain) was grown at 30 °C in YPD medium, harvested by centrifugation, washed extensively in PBS, and heated at 70 °C for 1 h, and 10^8^ organisms were injected IP (in 1 ml of PBS). Cohorts of mice were also injected with saline or PBS as a steady-state control.

Peripheral blood was obtained from the tail veins every 2 weeks. The mice were sacrificed if Hgb was <6.0 or platelets were <70,000. The mice were considered to have AML if the WBC count was >50,000, and circulating myeloid blasts were >30%.

Six mice were studied per group for an 80% chance of detecting a 40% difference between groups. Standard errors were comparable between groups. No mice were excluded from evaluation. Randomization and “blinding” were not required. Variance within groups was consistent in the various experimental cohorts.

### Chemotherapy treatment

4 weeks post-transplant, secondary recipients were treated with cytosine arabinoside (100 mg/kg IP on days 1–5) and doxorubicin (3 mg/kg IP on days 1–3) ± nintedanib (30 mg/kg IP starting day 1 and continuing until death), nintedanib only, or buffer control (30, 32). The mice were sacrificed for overwhelming AML (WBC > 100,000 or organomegaly) or at 40 weeks.

Ten mice were studied per group for an 80% chance of detecting a 20% difference between groups. Standard errors were comparable between groups. No mice were excluded from evaluation. Randomization and blinding were not required. Variance within groups was consistent.

### Human bone marrow studies

LIN^−^CD34^+^ cells were isolated from bone marrow of AML or control subjects using a magnetic bead–linked antibody system (Miltenyi Biotechnology, Auburn, CA, USA) (18, 24). RNA was isolated using TRIzol reagent.

### Quantitative real-time PCR

Primers were designed with Applied Biosystems software, and PCR was performed using the SYBR green “standard curve” method. The results were normalized to β-actin and 18S. Each independent sample was assayed in triplicate. Biological replicates exhibited a normal distribution.

### RNA-Seq and transcriptome analysis

Stranded mRNA-Seq was conducted in the Northwestern University NUSeq Core with RNA from LIN^−^ murine bone marrow cells (four per cohort with nonpooled samples). RNA quality was determined using an Agilent Bioanalyzer 2100 (Agilent Research Laboratories, Santa Clara, CA, USA). Sequencing libraries were prepared with the TruSeq Stranded mRNA kit (per manufacturer's instructions; Illumina Inc., San Diego, CA, USA) and validated. Single-end, 75-bp reads were generated using the lllumina NextSeq 500 Sequencer.

DNA read quality was evaluated using FastQC. Adapters were trimmed, and reads of poor quality or aligning to rRNA sequences were filtered. Cleaned reads were aligned to the *Mus musculus* genome using STAR, and read counts were calculated using htseq-count in conjunction with the mm10 gene annotation file (RRIDS:SCR_005780). Differential expression was determined using DESeq2 (37). Statistical significance of differentially expressed genes was a false discovery rate–adjusted *p* value of <0.05.

### RAP1 assays

Studies were performed with RAP1 activity kit per the manufacturer's instructions (16120; Thermo Scientific). A representative blot for three independent experiments is shown.

### Sternal bone marrow histology

Sternal bone marrow samples were fixed in 10% buffered formalin; decalcified in 10% formic acid, 5% formaldehyde; and embedded in paraffin; and a 4-μm section was cut. For histology, the sections were stained using hematoxylin and eosin (by the Pathology Core Facility of the Lurie Cancer Center). Light microscopy was performed, and digital images were captured (40× magnification).

### Sternal bone marrow immunohistochemistry

Hydrated, deparaffinized tissue was blocked with donkey serum and incubated with primary antibodies (1:100 dilution of anti-FGFR1 (LS-B6232) or anti-phospho-FGFR1 (LS-C96872) (LifeSpan Bioscience, Seattle, WA, USA) in 1% goat serum. Fluorescent secondary antibodies were applied (1:100 dilution of antibody conjugated with Alexa Fluor 488 or Alexa Fluor 546) (Invitrogen). Images were captured in a laser scanning confocal microscope at 60× magnification.

### Statistical analysis

Significance was determined by two-sided Student's *t* test or ANOVA using Sigmaplot or Sigmastat software. Survival curves were determined by Mann–Whitney rank sum test. *Error bars* represent the standard error, and *p* < 0.05 was considered significant.

### Animal subject approval

Research with animal studies was approved by the animal care and use committees of Northwestern University and Jesse Brown Veterans Affairs Medical Center.

### Human subject approval

Human subject studies abide by the Declaration of Helsinki principles. Human subject research was approved by the Institutional Review Boards of Northwestern University and Jesse Brown VAs.

### Data availability

Data are available upon request to the corresponding author.
